# Thrombosis and Major Bleeding Risk After Primary PCI Among Patients With Multivessel Coronary Artery Disease

**DOI:** 10.3389/fcvm.2021.729432

**Published:** 2022-02-08

**Authors:** Xiaoxiao Zhao, Chen Liu, Peng Zhou, Zhaoxue Sheng, Jiannan Li, Jinying Zhou, Runzhen Chen, Ying Wang, Yi Chen, Li Song, Hanjun Zhao, Hongbing Yan

**Affiliations:** ^1^Department of Cardiology, National Center for Cardiovascular Diseases, Peking Union Medical College, Fuwai Hospital, Chinese Academy of Medical Sciences, Beijing, China; ^2^Fuwai Hospital, Chinese Academy of Medical Sciences, Shenzhen, China

**Keywords:** thrombosis, major bleeding, primary PCI, multi-vessels coronary artery disease, inflammation

## Abstract

**Background and Aim:**

This study aimed to develop and validate separate risk prediction models for thrombosis events (TEs) and major bleeding (MB) in patients with multivessel coronary artery lesions who had undergone primary percutaneous coronary intervention (PCI).

**Methods and Results:**

Thrombosis events (TEs) were defined as the composite of myocardial infarction recurrence or ischemic cerebrovascular events, whereas MB was defined as the occurrence of bleeding academic research consortium (BARC) three or five bleeding. The derivation and validation cohorts comprised 2,976 patients who underwent primary PCI between January 2010 and June 2017. At a median follow-up of 3.07 years (1,122 days), TEs and MB occurred in 167 and 98 patients, respectively. Independent predictors of TEs were older age, prior PCI, non-ST elevated MI (NSTEMI), and stent thrombosis (ST). Independent predictors of MB were triple therapy at discharge, coronary artery bifurcation lesions, lesion restenosis, target lesion of the left main coronary artery, stent thrombosis, non-use of IABP during primary PCI, type A/B according to the American College of Cardiology classification of the coronary lesion, and PTCA. In the derivation and validation cohorts, the areas under the curve were 0.817 and 0.82 for thrombosis and 0.886 and 0.976 for bleeding, respectively. In the derivation cohort, high thrombotic risk (*n* = 755) was associated with higher 3-year incidence of TEs, major adverse cardiovascular events (MACEs), and all-cause death compared to low risk (*n* = 1,275) (*p* = 0.0022, 0.019, and 0.012, respectively). High bleeding risk (*n* = 1,675) was associated with higher incidence of bleeding, MACEs, and cardiac death compared to low risk (*n* = 355) (*p* < 0.0001).

**Conclusion:**

Simple risk scores can be useful in predicting risks of ischemic and bleeding events after primary PCI, thereby stratifying thrombotic or MB risks and facilitating clinical decisions.

## Introduction

The risk of thrombotic events (TEs), such as myocardial infarction (MI) and stent thrombosis (ST), is lower in patients with the acute coronary syndrome who receive dual antiplatelet therapy (DAPT) with aspirin or who have undergone primary percutaneous coronary intervention (PCI) (1). The marginally higher mortality associated with bleeding events has been reported to be comparable to the risk associated with MI (2, 3). Hence, avoiding bleeding events is becoming increasingly important. These results suggest that clinical decision-making concerning the optimal duration of DAPT for individual patients following PCI must be predicated on balancing the long-term risks of intensive antithrombotic therapy and avoiding major bleeding (MB) (4, 5). In this context, it is essential to develop stratification tools for distinguishing high-risk ischemic patients from high-risk bleeding patients. To date, most post-PCI algorithms are single scoring systems or focused on in-hospital events or short-term risks (6–11). Although it is practicable to inform clinical decisions with respect to the short-term provision of DAPT, respective weights of underlying risk factors vary from early as opposed to later, and predicting risks of thrombotic and bleeding events is more important in the long term. Accordingly, this study aimed to explore prediction rules for long-term outcomes of TEs and MB events separately in a large Chinese observational database of patients with multivessel coronary artery lesions who had undergone primary PCI. We prepared and presented the current article in accordance with the TRIPOD reporting checklist (Appendix File)^1^.

## Materials and Methods

### Study Population: Enrollment and Randomization

A prospective observational study was conducted on patients who had undergone primary PCI in Fuwai Hospital (National Center for Cardiovascular Diseases, Peking Union Medical College and Chinese Academy of Medical Sciences) in Beijing, China, between January 2010 and June 2017. This study was designed to investigate the validity of separate risk prediction models for subsequent clinical adverse events. With respect to eligibility criteria, adult patients (1) who had undergone primary PCI, including stent implantation, thrombus aspiration, and balloon dilation in the coronary artery, and (2) who provided written informed consent were included in the study. Patients (1) who refused participation, (2) who were lost to follow-up when contacted, and (3) who did not have coronary angiography parameters (coronary angiography, used to diagnose ischemic heart disease after chest pain, is a procedure that uses contrast dye and x-ray pictures to detect blockages in coronary arteries; the coronary angiography parameters mentioned above including whether patients used thrombus aspiration, stent implantation, use of IABP, percutaneous transluminal coronary angioplasty, and complex procedure) or multivessel coronary artery lesions were, however, excluded from the analysis. Following the application of the inclusion and exclusion criteria, a total of 3,976 subjects with acute myocardial infarction with multivessel lesions remained. The patients were administered aspirin 300 mg, clopidogrel 600 mg or ticagrelor 180 mg, and heparin 100 IU/kg before the procedure of intervention. The access of primary PCI was performed *via* radial or femoral artery. Thrombus aspiration was performed to reduce the burden of the thrombus. Duration of dual antiplatelet therapy (DAPT) consisted of oral aspirin and a P2Y12 inhibitor for at least 12 months following primary PCI.

The included patients were randomly and proportionally (70:30%) divided into the derivation cohort (*n* = 2,084) and the validation cohort (*n* = 892) (divide the training dataset into training and validation sets, ideally 7,030, and model on 70% of the training dataset; then, use the 30% validation data set for cross-validation and performance evaluation using evaluation metrics).

This study was conducted according to the principles outlined in the Declaration of Helsinki and was approved by the Ethics Committee of Fuwai Hospital. All the study subjects gave informed consent.

### Study Definitions

Thrombosis events (TEs) were defined as the occurrence of coronary thrombotic complications such as ischemic cerebrovascular events or MI recurrence. Consistent with the universal definition, MI recurrence was defined as the recurrence of chest pain accompanied by either re-ST-segment elevation or ST-segment depression attributed to myocardial ischemia and re-elevation of cardiac troponin I >25% (12). MB was defined as the occurrence of Bleeding Academic Research Consortium type three or five bleeding (13) and was adjusted by a blinded committee. Multivessel coronary artery lesions, characterized by significant stenosis (diameter ≥1.5 mm and significant stenosis >50%) in all three major coronary arteries determined by a cardiologist. Stroke was defined as rapidly developing focal or general brain dysfunction that lasted for more than 24 h or caused death, excluding non-vascular causes (e.g., trauma, metabolic disorders, tumors, and any neurological abnormalities due to central nervous system infection). Additionally, ischemic stroke included cerebral thrombosis and cerebral embolism. Imaging data are as follows: computed tomography (CT) showed insular signs, namely, disappearance and blurring of the gray matter interface in the conduction zone, consistency of the density of the insular cortex with the outer capsule, and disappearance or narrowing of the cerebral sulci in the cortex; the abnormal high signal shadow was found in the responsible lesion area under magnetic resonance imaging (MRI) detection; major adverse cardiovascular events (MACEs) were identified as the composite of overall mortality, MI recurrence, and ischemic cerebrovascular events; events were identified using physician-reported diagnoses extracted from cardiac catheterization laboratory report, hospital discharge records, or clinical notes in the event of death. Anemia was defined as hemoglobin level <12 g/dl in men and <11 g/dl in women (14). Complex PCI procedures included bifurcation, total occlusion, thrombus, or >2 stent. Chronic kidney disease (CKD) was defined as chronic renal structural and functional impairments due to a variety of causes such as eGFR lesions (<60 ml/min·1.73 m^2^), imaging abnormalities, and abnormal blood/urine composition for at least the past 3 months. The estimated glomerular filtration rate (eGFR) was calculated by the formula of Modification of Diet in Renal Disease (MDRD). No-reflow phenomenon was defined as Thrombolysis in Myocardial Infarction flow grade <3 after primary PCI. The patients were subsequently divided into the low-risk and high-risk groups according to the best cutoff value of the prognostic index. Triple therapy on discharge was defined as a combination of DAPT (aspirin plus thienopyridine) and oral anticoagulation therapy.

### Follow-Up

The patients were followed-up at least 1 year after discharge by physicians. The health status of the enrolled patients was confirmed *via* telephone calls and review of health records, and this method, which was approved by the Review Board of Fuwai Hospital. The follow-up primary endpoints including TEs, MB, MACEs, cardiac death, and all cause death, were identified and extracted primary endpoints from recordings of hospital records, laboratory reports, and clinical notes in the event of death by physicians who in charge of follow-up.

### Statistical Analysis

Normal distribution of outcome variables was conducted using the method of Kolmogorov–Smirnov test. Baseline clinical and procedural characteristics of primary PCI according to the presence or absence of TEs and MB were compared between patients with and without multivessel lesions, and between the derivation and validation cohorts by chi-square test and Student's *t*-test for categorical variables and continuous variables, respectively. Cox proportional hazards regression and least absolute shrinkage and selection operator (LASSO) regression generated separate prediction models to achieve reduction and simplification of the models and to prevent the occurrence of overfitting, with time to the first occurrence of TEs or MB serving as the dependent variable in each respective model. Event-free patients were censored at the end of the study, or at the time of death or last contact, whichever came first.

To account for missing data on serum low-density lipoprotein cholesterol at baseline (*n* = 9, 0.4%), history of smoking (*n* = 192, 9.2%), body mass index (*n* = 114, 5.5%), and prior hyperlipidemia (*n* = 210, 10.1%), covariates for each model were identified through an iterative process involving multiple imputation with automated variable selection. Five imputations were generated from the original dataset, and multivariate normal regression was used to substitute the missing data on serum low-density lipoprotein cholesterol, history of smoking, body mass index, and prior hyperlipidemia within each impute in the first step.

Candidate covariates for each model were age, sex, triple therapy at discharge, body mass index (<25, 25–35 [reference], >35 kg/m^2^), smoking status, prior hyperlipidemia, hypertension, diabetes mellitus, prior coronary artery bypass grafting, prior PCI, CKD, abnormal liver function, malignancy, Killip classification, length of the lesion, complex procedure (bifurcation, total occlusion, thrombus, or >2 stents), anemia, eGFR <60 ml/min, platelet count <1,00,000/μl, percutaneous transluminal coronary angioplasty, the target of bifurcation, use of intra-aortic balloon pump (IABP), stent implantation, no-reflow phenomenon, revascularization after discharge, lesion restenosis, and target lesion of the left main, right, left circumflex, or left anterior descending coronary artery. Finally, one imputes with regression coefficients combined across all imputed datasets, as described by Rubin, was generated using the covariates (15). Variables that remained significant at a threshold of min Se were retained as final predictors by LASSO regression. Model discrimination was quantified using Harrell's c-statistic and calibration chart for the derivation and validation cohorts. At the beginning of the model establishment of LASSO regression, all identified independent variables were selected to minimize model deviation caused by the non-inclusion of significant independent variables, which were selected by univariable regression. Therefore, multivariable regression was not conducted. To improve prediction accuracy, the established model needs to find the set of independent variables with the strongest explanatory power for the dependent variables. A more refined model is obtained by constructing a function that compresses some coefficients and sets some coefficients to 0, 0.5, or minimization. LASSO regression is a biased estimation of data with complex collinearity retaining the advantage of contraction. The Lars algorithm software package of R language provided LASSO programming. Hence, variable selection and dimensionality reduction can be achieved accurately by LASSO regression.

Integer risk scores for the outcomes of TEs and MB were generated using fully adjusted regression coefficients, as described by Sullivan et al. (16). The survival receiver operating characteristic (ROC) curve for prognostic index (PI) was fitted using the Kaplan–Meier method, and the best cutoff value was obtained. The patients were subsequently divided into the low-risk and high-risk groups according to the best cutoff value of the prognostic index (PI). Observed event rates were calculated as Kaplan–Meier (K-M) estimates of time to the first event. Predicted event rates were estimated using fully adjusted Cox regression models. The main software used for statistical analysis in this study used survival and rms package in R language version I 386 3.6.3. Other analyses were performed using SPSS version 20.0 (IBM Corp., Armonk, NY, United States). All *p*-values were two-tailed, and statistical significance was set at *p* < 0.05.

### Performance and Internal Validation Cohort

External validation of each score was performed. Each subject in the validation cohort was assigned with a TE risk score and MB event risk score in the same manner as in the derivation cohort. The patients were subsequently categorized into subjects with low and high thrombotic and bleeding risks using the same thresholds as in the derivation cohort. The 3-year adverse event rates were counted for each risk division using the K-M method, and the difference was determined by the method of the log-rank test. Discriminations of both the derivation and validation cohorts were assessed by calculating the area under the ROC curve (AUC) and expressed as c-statistic with the use of the MedCalc software for Windows, version 18.2.1.0 (MedCalc Software, Mariakerke, Belgium). The accuracy of the new model and acuity (bleeding model)/autar (thrombosis model) risk score model predicting MACEs among patients with MI who underwent PPCI was compared according to the area under the ROC (AUC) curve by a non-parametric test developed. MedCalc for Windows version 18.2.1 (MedCalc Software, Mariakerke, Belgium) was used for comparison.

## Results

### Baseline Characteristics

Among 4,151 enrolled patients who had undergone PCI in Fuwai Hospital (Beijing, China) between January 2010 and June 2017, those who were lost to follow-up (*n* = 97), had no coronary angiography parameters (*n* = 78), and had no multivessel coronary artery lesions (*n* = 1,000) were excluded. Hence, the study population comprised 2,976 patients in total. At a median follow-up of 3.07 years (1,122 days), 167 patients sustained TEs, whereas 98 patients experienced MB.

Of the patients suffering MB, the incidence of cerebral hemorrhage, fundus bleeding, gastrointestinal bleeding, urogenital bleeding, nasal mucosa bleeding was 11.22 (11), 19.39 (19), 53.06 (52), 7.14 (7), and 9.18% (9), respectively. The baseline characteristics are summarized in Tables 1.1, 1.2. Patients with TEs more frequently presented with MACEs (*p* < 0.001) and showed a higher incidence of hyperlipidemia (*p* = 0.017) than those without TEs. Patients with MB had lower degree of lesion stenosis (*p* = 0.034) and showed higher incidence of all-cause death (*p* = 0.033), cardiac death (*p* = 0.041), and cerebral hemorrhage (*p* < 0.001) than their counterparts without MB.

**Table 1.1:** Baseline clinical characteristics of patients with vs. without thrombotic events or major bleeding events.

**Variables**	**Multi-vessels coronary artery disease (*****N*** **=** **2,976)**					**Non muti-vessels coronary artery disease (*****N*** **=** **1,000)**				
	**TEs (*N* = 167)**	**No TEs (*N* = 2,809)**	**P_**1**_**	**MB (*N* = 98)**	**No MB (*N* = 2,878)**	**P_**2**_**	**TEs (*N* = 31)**	**No TEs (*N* = 969)**	**MB (*N* = 24)**	**No MB (*N* = 976)**
Age (years)	61.56 ± 0.84	60.21 ± 0.22	0.137	60.03 ± 1.16	60.30 ± 0.21	0.821	59.10 ± 2.04	55.04 ± 0.40	57.0 ± 2.49	55.1 ± 0.40
Male [%(*n*)]	131 (78.4%)	2,185 (77.8%)	0.465	78 (79.59%)	2,238 (77.76%)	0.668	24 (77.4%)	792 (81.0%)	20 (83.3%)	796 (81.6%)
BMI (kg/m^2^)	26.06 ± 0.39	25.92 ± 0.07	0.627	26.74 ± 0.51	25.91 ± 0.07	0.480	25.35 ± 0.58	25.99 ± 0.12	26.3 ± 0.62	26.0 ± 0.12
Heart rate (beats/min)	74.61 ± 1.26	77.54 ± 0.30	0.023^*^	78.69 ± 1.69	77.33 ± 0.30	0.407	65.06 ± 4.86	68.97 ± 0.66	63.2 ± 5.32	69.0 ± 0.66
SBP (mmHg)	125.51 ± 1.54	123.56 ± 0.36	0.539	124.46 ± 2.21	124.61 ± 0.36	0.937	129.16 ± 3.7	122.85 ± 0.56	119.6 ± 3.3	123.1 ± 0.6
DBP (mmHg)	69.42 ± 1.39	71.05 ± 0.31	0.223	72.78 ± 1.46	70.90 ± 0.31	0.267	75.26 ± 2.32	74.71 ± 0.31	70.3 ± 2.19	74.8 ± 0.41
LVEF at admission	52.82 ± 0.59	54.11 ± 0.29	0.291	54.31 ± 0.85	54.03 ± 0.29	0.855	54.19 ± 1.36	53.53 ± 0.24	54.8 ± 1.47	53.5 ± 0.24
Hypertension [%(*n*)]	109 (65.3%)	1,805 (64.3%)	0.431	66 (67.35%)	1,848 (64.21%)	0.524	18 (58.1%)	493 (50.9%)	13 (54.2%)	498 (51.0%)
Diabetes [%(*n*)]	64 (38.3%)	991 (35.3%)	0.236	32 (32.65%)	1,023 (35.55%)	0.556	10 (32.3%)	237 (24.5%)	8 (33.3%)	239 (24.5%)
Hyperlipidemia [%(*n*)]	143 (87.2%)	2,329 (92.4%)	0.017^*^	79 (91.86%)	2,393 (92.07%)	0.943	25 (80.6%)	796 (93.3%)	19 (79.2%)	802 (93.3%)
Smoking [%(*n*)]	107 (65.2%)	1,654 (65.1%)	0.521	60 (69.77%)	1,701 (64.87%)	0.349	22 (71.0%)	573 (66.8%)	15 (62.5%)	580 (67.1%)
Previous PCI [%(*n*)]	27 (16.2%)	411 (14.6%)	0.326	17 (17.35%)	421 (14.63%)	0.456	3 (9.7%)	106 (10.9%)	2 (8.3%)	107 (11.0%)
Previous CABG [%(*n*)]	5 (3.0%)	36 (1.3%)	0.077	2 (2.04%)	39 (1.36%)	0.567	4 (0.4%)	0 (0.0%)	4 (0.4%)	0 (0.0%)
AF [%(*n*)]	14 (8.4%)	169 (6.0%)	0.143	6 (6.12%)	177 (6.15%)	0.991	4 (12.9%)	52 (5.4%)	2 (8.3%)	54 (5.5%)
CKD [%(*n*)]	14 (8.4%)	237 (8.4%)	0.561	10 (10.20%)	241 (8.37%)	0.521	0 (0.0%)	52 (5.4%)	2 (8.3%)	50 (5.1%)
HDL (mg/dl)	1.53 ± 0.08	1.70 ± 0.02	0.802	1.81 ± 0.10	1.69 ± 0.02	0.328	1.65 ± 0.26	1.74 ± 0.04	1.87 ± 0.47	1.73 ± 0.04
LDL (mg/dl)	2.67 ± 0.07	2.74 ± 0.02	0.381	2.67 ± 0.09	2.74 ± 0.02	0.526	2.74 ± 0.19	2.76 ± 0.03	2.58 ± 0.21	2.76 ± 0.03
Triglycerides (mg/dl)	1.07 ± 0.02	1.05 ± 0.01	0.321	1.01 ± 0.02	1.05 ± 0.01	0.030^*^	1.13 ± 0.06	1.07 ± 0.01	1.00 ± 0.00	1.07 ± 0.01
LPA (g/L)	302.53 ± 22.74	271.20 ± 4.62	0.179	274.92 ± 24.8	272.89 ± 4.62	0.937	239 ± 39.08	249 ± 7.80	243.7 ± 42	249.1 ± 7.8
hs-CRP (mg/L)	7.49 ± 0.39	7.56 ± 0.09	0.684	7.22 ± 0.51	7.65 ± 0.09	0.402	9.45 ± 1.06	7.35 ± 0.16	7.63 ± 1.27	7.41 ± 0.16
D-dimer of baseline (ug/L)	0.72 ± 0.26	0.57 ± 0.03	0.552	0.44 ± 0.11	0.58 ± 0.04	0.455	0.37 ± 0.09	0.49 ± 0.05	0.21 ± 0.10	0.50 ± 0.05
Peak level of D-dimer (ug/L)	1.22 ± 0.38	0.92 ± 0.05	0.437	0.94 ± 0.25	0.94 ± 0.05	0.999	0.72 ± 0.18	0.97 ± 0.10	0.57 ± 0.21	0.97 ± 0.10
Crea (umol/L)	80.24 ± 1.76	82.75 ± 0.49	0.219	85.81 ± 3.24	82.50 ± 0.47	0.209	79.58 ± 3.19	79.34 ± 0.63	82.00 ± 3.8	79.28 ± 0.6
eGFR (MDRD) (ml/min per 1.73 m^2^)	99.32 ± 8.28	88.61 ± 1.56	0.205	88.49 ± 7.43	89.24 ± 1.58	0.931	85.97 ± 3.96	93.58 ± 2.60	84.71 ± 3.9	93.56 ± 2.6
Peak level of TnI (ng/L)	2.22 ± 0.81	4.05 ± 0.27	0.033^*^	3.96 ± 1.69	3.95 ± 2.26	0.996	6.83 ± 4.33	3.87 ± 0.42	8.35 ± 4.93	3.83 ± 0.41
Statin [%(*n*)]	156 (93.4%)	2,627 (93.5%)	0.526	95 (96.94%)	2,688 (93.40%)	0.162	30 (96.8%)	909 (93.8%)	22 (91.7%)	917 (94.0%)
Aspirin [%(*n*)]	166 (99.4%)	2,780 (99.0%)	0.491	97 (98.98%)	2,849 (98.99%)	0.990	29 (93.5%)	961 (99.2%)	24 (100%)	966 (99.0%)
Clopidogrel [%(*n*)]	148 (88.6%)	2,145 (76.4%)	<0.001^*^	71 (72.45%)	2,222 (77.21%)	0.271	28 (90.3%)	731 (75.4%)	23 (95.8%)	736 (75.4%)
Ticagrelor [%(*n*)]	19 (11.4%)	643 (23.1%)	<0.001^*^	27 (27.55%)	635 (22.23%)	0.214	2 (6.5%)	229 (23.7%)	1 (4.2%)	230 (23.7%)
ACEI [%(*n*)]	106 (63.5%)	1,710 (60.9%)	0.280	62 (63.27%)	1,754 (60.95%)	0.643	15 (48.4%)	625 (64.5%)	18 (75.0%)	622 (63.7%)
ARB [%(*n*)]	14 (8.4%)	250 (8.9%)	0.479	8 (8.16%)	256 (8.90%)	0.802	4 (12.9%)	82 (8.5%)	1 (4.2%)	85 (8.7%)
ACEI/ARB [%(*n*)]	120 (71.9%)	1,957 (69.7%)	0.307	70 (71.43%)	2,007 (69.74%)	0.720	19 (61.3%)	707 (73.0%)	19 (79.2%)	707 (72.4%)
Beta-Blockers [%(*n*)]	148 (88.6%)	2,430 (86.5%)	0.258	82 (83.67%)	2,496 (86.73%)	0.383	26 (83.9%)	865 (89.3%)	16 (66.7%)	875 (89.7%)
Diuretic [%(*n*)]	53 (31.7%)	794 (28.3%)	0.189	25 (25.51%)	822 (28.56%)	0.510	10 (32.3%)	293 (30.2%)	6 (25.0%)	297 (30.4%)
Spironolactone [%(*n*)]	41 (24.6%)	575 (20.5%)	0.123	16 (16.33%)	600 (20.85%)	0.277	9 (29.0%)	240 (24.8%)	7 (29.2%)	242 (24.8%)
P2Y12 inhibitors	167 (100%)	2,787 (99.2%)	0.279	98 (100.00%)	2,856 (99.24%)	0.385	30 (96.8%)	960 (99.1%)	24 (100%)	966 (99.0%)
Total lesion length, mm	28.37 ± 1.25	28.47 ± 0.31	0.937	28.73 ± 1.67	28.45 ± 0.30	0.865	22.03 ± 1.54	23.99 ± 0.42	19.96 ± 1.9	24.03 ± 0.4
Lesion diameter, mm	3.20 ± 0.05	3.13 ± 0.01	0.197	3.21 ± 0.07	3.13 ± 0.01	0.263	3.32 ± 0.15	3.29 ± 0.02	3.46 ± 0.15	3.29 ± 0.02
Degree of lesion stenosis	97.10 ± 0.43	97.22 ± 0.11	0.784	95.85 ± 0.65	97.26 ± 0.11	0.034^*^	97.90 ± 1.15	97.07 ± 0.18	97.67 ± 1.2	97.08 ± 0.2
Bifurcation lesion [%(*n*)]	59 (35.3%)	984 (35.0%)	0.499	34 (34.69%)	1,009 (35.06%)	0.941	9 (29.0%)	304 (31.4%)	5 (20.8%)	308 (31.6%)
PTCA [%(*n*)]	144 (86.2%)	2,512 (89.4%)	0.123	93 (94.90%)	2,563 (89.05%)	0.066	24 (77.4%)	817 (84.3%)	18 (75.0%)	823 (84.3%)
Thrombus aspiration [%(*n*)]	55 (32.9%)	1,148 (40.9%)	0.025^*^	37 (37.76%)	1,166 (40.51%)	0.584	16 (51.6%)	443 (45.7%)	13 (54.2%)	446 (45.7%)
Stent implantation [%(*n*)]	145 (86.8%)	2,479 (88.3%)	0.325	88 (89.80%)	2,536 (88.12%)	0.613	26 (83.9%)	856 (88.3%)	22 (91.7%)	860 (88.1%)
IABP [%(*n*)]	17 (10.2%)	287 (10.2%)	0.558	5 (5.10%)	299 (10.39%)	0.089	4 (12.9%)	79 (8.2%)	5 (20.8%)	78 (8.0%)
MACE [%(*n*)]	166 (99.4%)	171 (6.1%)	<0.001^*^	7 (7.14%)	330 (11.47%)	0.184	31 (100%)	27 (2.8%)	4 (16.7%)	54 (5.5%)
All caused mortality [%(*n*)]	10 (6.0%)	171 (6.1%)	0.562	1 (1.02%)	180 (6.25%)	0.033^*^	3 (9.7%)	27 (2.8%)	3 (12.5%)	27 (2.8%)
Cardiovascular death [%(*n*)]	7 (4.2%)	111 (4.0%)	0.497	0 (0.00%)	118 (4.10%)	0.041^*^	1 (3.2%)	18 (1.9%)	0 (0.0%)	19 (1.9%)
Recurrence MI [%(*n*)]	111 (66.5%)	0 (0.0%)	<0.001^*^	5 (5.10%)	106 (3.69%)	0.468	18 (58.1%)	0 (0.0%)	0 (0.0%)	18 (1.8%)
Ischemic stroke [%(*n*)]	58 (34.7%)	0 (0.0%)	<0.001^*^	2 (2.04%)	56 (1.95%)	0.847	13 (41.9%)	0 (0.0%)	3 (12.5%)	10 (1.0%)
Cerebral hemorrhage [%(*n*)]	0 (0.0%)	11 (0.4%)	0.529	11 (11.22%)	0 (0.00%)	<0.001^*^	2 (6.5%)	1 (0.1%)	3 (12.5%)	0 (0.0%)

*Values are expressed as mean ± standard error or number (%). BMI, body mass index; SBP, systolic blood pressure; DBP, diastolic blood pressure; LVEF, left ventricular ejective fraction; PCI, percutaneous coronary intervention; CABG, coronary artery bypass grafting; CKD, chronic kidney disease; HDL-C, high-density lipoprotein cholesterol; LDL-C, low-density lipoprotein cholesterol; TG, triglyceride; LPA, lipase activator; hs-CRP, high sensitive C-reactive protein; eGFR, estimated glomerular filtration rate; ACEI, angiotensin-converting enzyme inhibitor; ARB, angiotensin receptor blocker; MACE, major adverse cardiovascular event; TEs, thrombotic events; MB, major bleeding*.

* *p < 0.05*.

**Table 1.2:** Baseline clinical characteristics of subgroup patients with vs. without thrombotic events or major bleeding. events.

**Variables**	**Derivation cohort**		**Validation cohort**		**P_**1**_**	**P_**2**_**
	**TEs**	**MB**	**TEs**	**MB**		
Age (years)	61.68 ± 1.12	59.75 ± 1.24	61.05 ± 1.27	60.69 ± 2.61	0.626	0.714
Male [%(*n*)]	79 (77.5%)	57 (82.6%)	52 (80.0%)	21 (72.4%)	0.425	0.191
Hypertension [%(*n*)]	65 (63.7%)	43 (62.3%)	44 (67.7%)	23 (79.3%)	0.361	0.078
Diabetes [%(*n*)]	39 (38.2%)	23 (33.3%)	25 (38.5%)	9 (31.0%)	0.552	0.510
Hyperlipidemia [%(*n*)]	86 (86.9%)	58 (93.5%)	57 (87.7%)	21 (87.5%)	0.539	0.301
Smoking [%(*n*)]	66 (66.0%)	45 (72.6)	41 (63.1%)	15 (62.5%)	0.413	0.255
Previous PCI [%(*n*)]	18 (17.6%)	11 (15.9%)	9 (13.8%)	6 (20.7%)	0.335	0.383
Previous CABG [%(*n*)]	2 (2.0%)	1 (1.4%)	3 (4.6%)	1 (3.4%)	0.297	0.506
Atrialfibrillation [%(*n*)]	11 (10.8%)	5 (7.2%)	3 (4.6%)	1 (3.4%)	0.131	0.424
CKD [%(*n*)]	10 (9.8%)	7 (10.1%)	4 (6.2%)	3 (10.3%)	0.299	0.616
**Laboratory examinations**
HDL <0.7 (mg/dl)	9 (8.8%)	6 (8.7%)	8 (12.3%)	5 (17.2%)	0.318	0.189
LDL>3.12 (mg/dl)	25 (24.5%)	20 (29.0%)	21 (32.3%)	6 (20.7%)	0.178	0.279
Triglycerides>1.7 (mg/dl)	3 (2.9%)	1 (1.4%)	2 (3.1%)	0 (0.0%)	0.647	0.704
LPA>300 (g/L) [%(*n*)]	38 (37.3%)	24 (34.8%)	22 (22.8%)	9 (31.0%)	0.390	0.455
hs-CRP>10(mg/L) [%(*n*)]	39 (38.2%)	28 (40.6%)	31 (47.7%)	11 (37.9%)	0.148	0.495
D-dimer>0.5(ug/L) [%(*n*)]	23 (22.5%)	14 (20.3%)	10 (15.4%)	9 (31.0%)	0.175	0.187
eGFR <90(ml/min per 1.73 m^2^) [%(*n*)]	61 (59.8%)	35 (50.7%)	42 (64.6%)	20 (69.0%)	0.324	0.074
**Procedural characteristics**
PTCA [%(*n*)]	88 (86.3%)	67 (97.1)	56 (86.2)	26 (89.7%)	0.578	0.152
Thrombus aspiration [%(*n*)]	36 (35.3%)	22 (31.9%)	19 (29.2%)	15 (51.7%)	0.261	0.053
Stent implantation [%(*n*)]	89 (87.3%)	61 (88.4%)	56 (86.2%)	27 (93.1%)	0.507	0.384
Use of IABP [%(*n*)]	7 (6.9%)	3 (4.3%)	10 (15.4%)	2 (6.9%)	0.067	0.465
LM lesion [%(*n*)]	4 (3.9%)	9 (12.0%)	6 (9.2%)	2 (6.9%)	0.142	0.310
Complexprocedure (bifurcation, totalocclusion, thrombus) [%(*n*)]	88 (86.3%)	55 (79.7%)	54 (83.1%)	25 (86.2%)	0.362	0.326
Totalocclusion [%(*n*)]	67 (65.7%)	33 (47.8%)	42 (64.6%)	17 (58.6%)	0.508	0.226
Triple therapy on discharge	0 (0%)	2 (2.9%)	0 (0%)	0 (0%)	-	-
**Other characteristics**
Abnormal liverfunction [%(*n*)]	7 (6.9%)	3 (4.3%)	1 (1.5%)	2 (6.9%)	0.112	0.465
Malignancy [%(*n*)]	1 (1.0%)	1 (1.4%)	0 (0.0%)	1 (3.4%)	0.611	0.506
Anemia (Hb <11 g/dL) [%(*n*)]	3 (2.9%)	1 (1.4%)	1 (1.5%)	2 (6.9%)	0.493	0.208
Platelet count <100 000/lL [%(*n*)]	1 (1.0%)	1 (1.4%)	2 (3.1%)	0 (0.0%)	0.336	0.704

*Values are expressed as mean ± standard error or number (%). BMI, body mass index; SBP, systolic blood pressure; DBP, diastolic blood pressure; PCI, percutaneous coronary intervention; CABG, coronary artery bypass grafting; PTCA, percutaneous transluminal coronary angioplasty; CKD, chronic kidney disease; HDL-C, high-density lipoprotein cholesterol; LDL-C, low-density lipoprotein cholesterol; TG, triglyceride; LPA, lipase activator; hs-CRP, high sensitive C-reactive protein; eGFR, estimated glomerular filtration rate; ACEI, angiotensin-converting enzyme inhibitor; ARB, angiotensin receptor blocker; MACE, major adverse cardiovascular event; TEs, thrombotic events; MB, major bleeding*.

There was no significant difference in baseline characteristics between the derivation cohort and the validation cohort with respect to several aspects, such as clinical variables, laboratory examination, and procedural characteristics (Table 1.2). For the purpose of assessing the strength of association between 39 potential predictors and TEs or MB events in the derivation cohort, we constructed univariate Cox regression models and presented the results as hazard ratios (95% CI) with *p*-values. Variables identified to show association (with *p* < 0.5) in the univariate logistic regression models were included in the multivariate models (Table 2).

**Table 2:** Univariate Cox analysis for major bleeding events and thrombotic events in the derivation cohort.

**Variables**	**Univariate**	***P* Value**	**Univariate**	***P* Value**
	**HR (95% CI)**		**HR (95% CI)**	
	**Major bleeding events**		**Thrombotic events**	
Age, per year increase	1.000 (0.980, 1.021)	0.976	1.022 (1.005, 1.040)	0.013^*^
Male	1.332 (0.715, 2.483)	0.367^*^	0.919 (0.577, 1.464)	0.723
Triple therapy on discharge	15.525 (3.725, 64.242)	<0.001^*^	0.050 (0.000, 1.843)	0.839
BMI <25.0	1.079 (0.657, 1.773)	0.764	1.307 (0.872, 1.958)	0.194^*^
BMI>35.0	0.986 (0.159, 6.115)	0.987	1.320 (0.361, 4.829)	0.673
Hyperlipidemia	1.738 (0.627, 4.814)	0.287^*^	0.900 (0.494, 1.539)	0.730
Hypertension	0.902 (0.554, 1.468)	0.679	1.003 (0.667, 1.507)	0.988
Diabetes mellitus	1.030 (0.624, 1.700)	0.908	1.312 (0.877, 1.963)	0.187^*^
Current smoking	1.319 (0.768, 2.267)	0.316^*^	0.960 (0.635, 1.453)	0.848
Prior CABG	1.968 (0.272, 14.216)	0.502	3.721 (0.913, 15.157)	0.067^*^
Prior PCI	1.587 (0.828, 3.044)	0.164^*^	2.449 (1.458, 4.112)	0.001^*^
CKD	1.435 (0.656, 3.136)	0.366^*^	1.461 (0.760, 2.811)	0.256^*^
AF	1.061 (0.426, 2.642)	0.900	1.477 (0.787, 2.771)	0.225^*^
Abnormal liverfunction	0.619 (0.193, 1.979)	0.418^*^	1.509 (0.695, 3.280)	0.299^*^
Malignancy	1.894 (0.262, 13.685)	0.527	1.699 (0.236, 12.224)	0.599
KILLIP	ref	0.875	ref	0.931
KILLIP II	1.233 (0.611, 2.489)	0.559	0.906 (0.471, 1.745)	0.768
KILLIP III	1.046 (0.145, 7.556)	0.964	1.517 (0.373, 6.171)	0.560
KILLIP IV	0.567 (0.078, 4.101)	0.574	0.977 (0.309, 3.094)	0.969
Anemia (Hb <11 g/dL)	0.494 (0.068, 3.570)	0.485^*^	1.463 (0.461, 4.650)	0.519
Platelet count <100 000/lL	1.928 (0.267, 13.910)	0.515	1.424 (0.198, 10.231)	0.725
LDL-C(mmol/L) (>3.12)	0.882 (0.524, 1.485)	0.637	0.671 (0.426, 1.056)	0.084^*^
Hs-CRP (mg/L) (>10)	0.882 (0.545, 1.427)	0.609	0.742 (0.496, 1.110)	0.146^*^
HDL-C(mmol/L) (<0.7)	1.084 (0.468, 2.510)	0.851	1.252 (0.629, 2.493)	0.522
TG(mmol/L) (>1.7)	0.620 (0.086, 4.464)	0.635	1.269 (0.402, 4.006)	0.684
Lpa(mg/L) (>300)	1.176 (0.716, 1.933)	0.522	1.444 (0.964, 2.163)	0.075^*^
D2B time (>90 min)	1.014 (0.543, 1.895)	0.965	0.647 (0.402, 1.043)	0.074^*^
Target of LM	2.108 (1.045, 4.254)	0.037^*^	0.647 (0.238, 1.762)	0.395^*^
Target of RCA	1.081 (0.673, 1.735)	0.748	1.329 (0.838, 1.830)	0.282^*^
Target of LCX	1.418 (0.788, 2.550)	0.244^*^	1.184 (0.703, 1.995)	0.526
Target of LAD	1.518 (0.902, 2.555)	0.116^*^	0.776 (0.513, 1.172)	0.228^*^
Total lesion length, mm	ref	0.948	ref	0.599
20–40	0.937 (0.548, 1.603)	0.813	0.814 (0.524, 1.263)	0.358^*^
>40	1.031 (0.519, 2.049)	0.930	0.990 (0.571, 1.716)	0.971
Complexprocedure (bifurcation, totalocclusion, thrombus)	0.615 (0.341, 1.107)	0.105^*^	0.863 (0.490, 1.520)	0.609
Target ofbifurcation	1.405 (0.873, 2.262)	0.162^*^	0.809 (0.532, 1.231)	0.323^*^
PTCA	5.238 (1.282, 21.402)	0.021^*^	1.012 (0.573, 1.786)	0.968
Thrombus aspiration	0.736 (0.441, 1.228)	0.240^*^	0.971 (0.638, 1.479)	0.893
Stent implantation	0.806 (0.384, 1.693)	0.569	0.567 (0.314, 1.021)	0.059^*^
Use of IABP	0.394 (0.124, 1.255)	0.115^*^	0.624(0.289,1.345)	0.229^*^
No reflow phenomenon	0.361 (0.050, 2.603)	0.312^*^	0.941 (0.346, 2.560)	0.905
Revascularization after discharge	1.266 (0.646, 2.478)	0.492^*^	1.536 (0.898, 2.626)	0.117^*^
Restenosis of the lesion	5.624 (2.019, 15.669)	0.001^*^	1.607 (0.222, 11.646)	0.639
Stent thrombosis	0.048 (0.000, 29.282)	0.350^*^	2.771 (1.209, 6.352)	0.016^*^
Type of ACC = C1	0.575 (0.328, 1.007)	0.053^*^	0.483 (0.305, 0.765)	0.002^*^

*Values are expressed as mean ± standard error or number (%). BMI, body mass index; SBP, systolic blood pressure; DBP, diastolic blood pressure; PCI, percutaneous coronary intervention; CABG, coronary artery bypass grafting; CKD, chronic kidney disease; HDL-C, high-density lipoprotein cholesterol; LDL-C, low-density lipoprotein cholesterol; TG, triglyceride; LPA, lipase activator; hs-CRP, high sensitive C-reactive protein; eGFR, estimated glomerular filtration rate; ACEI, angiotensin-converting enzyme inhibitor; ARB, angiotensin receptor blocker; MACE, major adverse cardiovascular event; TEs, thrombotic events; MB, major bleeding*.

* *p < 0.5*.

### Predictors of Thrombotic Risk Scores and MB Risk Scores

Point estimates for each predictive covariate in the final prediction models are shown in Tables 3.1, 3.2. The strongest predictors of TEs, quantified and ranked using the values in the nomogram, were older age, history of prior PCI, non-ST-elevation MI, and stent thrombosis status. Correspondingly, the strongest contributors to MB were percutaneous transluminal coronary angioplasty, non-use of IABP during primary PCI, target lesion of the left main coronary artery, triple therapy at discharge, lesion restenosis, and type A/B according to the American College of Cardiology classification of coronary lesions, without stent thrombosis and coronary artery bifurcation lesions. The coronary TE prediction model had a moderate level of discrimination, with a c-index of 0.616 and adequate calibration for the entire population. Analogous parameters of model performance for MB in the entire cohort had a c-statistic of 0.676. Using the fully adjusted regression coefficients and nomogram graph, we developed integer-based risk projects for both MB and TEs (Tables 3.1, 3.2, respectively).

**Table 3.1:** Integer risk score for major bleeding.

**Integer risk score for major bleeding**		
**Parameter**	**Category of parameter**	**Score**
PTCA	YES	+30.8
	NO	+0
The use of IABP during primary PCI	YES	+0
	NO	+22.1
Target lesion of the LM coronary artery	YES	+16.8
	NO	+0
Triple therapy on discharge#	YES	+55
	NO	+0
Restenosis of the lesion	YES	+29.6
	NO	+0
C type of ACC classification of coronary	YES	+0
	NO	+8.25
Stent thrombosis	YES	+0
	NO	+100
Bifurcation lesions of coronary artery	YES	+6.47
	NO	+0
Total point: 0–240

*#Triple therapy on discharge was defined as a combination of dual antiplatelet therapy (DAPT) (aspirin plus thienopyridine) and oral anticoagulation therapy. PCI, percutaneous coronary intervention; PTCA, percutaneous transluminal coronary angioplasty; ACC, American College of Cardiology; LM, left main coronary artery*.

**Table 3.2:** Integer risk score for thrombotic events.

**Integer risk score for thrombotic events**		
**Parameter**	**Category of parameter**	**Score**
Age	<50 yer	+0
	50–59	+25
	60–69	+50
	70–79	+70
	≥80	+100
Prior PCI	YES	+78.1
	NO	+0
NSTEMI	YES	+66.6
	NO	+0
Stent thrombosis	YES	+0
	NO	+76.5
Total point: 0–350

*PCI, percutaneous coronary intervention; NSTEMI, non-ST-segment elevation myocardial infarction*.

**Figure 1:**
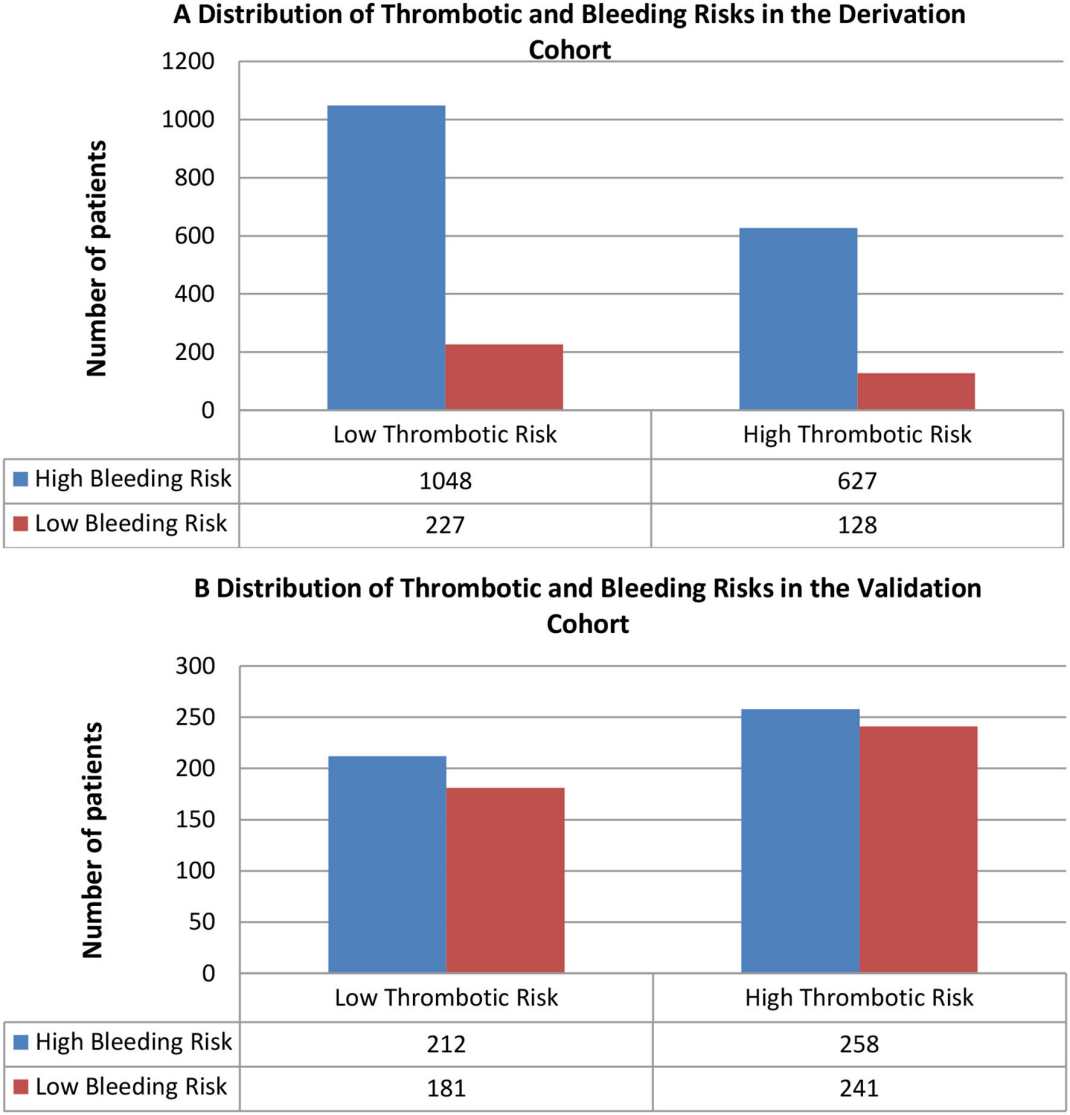
Distribution of bleeding risk score categories according to thrombotic risk score categories in the **(A)** derivation cohort (*N* = 2,030, *p* = 0.336) and **(B)** validation cohort (*N* = 892, *p* = 0.275). Blue represents high bleeding risk, and red represents low bleeding risk.

Furthermore, 627 (83%) out of 755 subjects with high TEs risk scores in the derivation cohort and 258 (51.7%) out of 499 subjects with high TEs risk scores in the validation cohort also had high MB risk scores; mortality and MB rates for these subjects were very high (Figure 1). Among those with high thrombotic risk scores, only 128 patients (17.0%) in the derivation cohort and 241 patients (48.3%) in the validation cohort had low bleeding risk scores (Figure 1). The majority of patients with low thrombotic risk scores had high bleeding risk scores (Figure 1). Within each thrombotic risk level, the frequency of high MB risk increased in the majority of patients as the thrombotic risk increased.

### Clinical Outcomes of Thrombosis and Clinically Relevant Bleeding in the Derivation and Validation Cohorts

**Figure 2:**
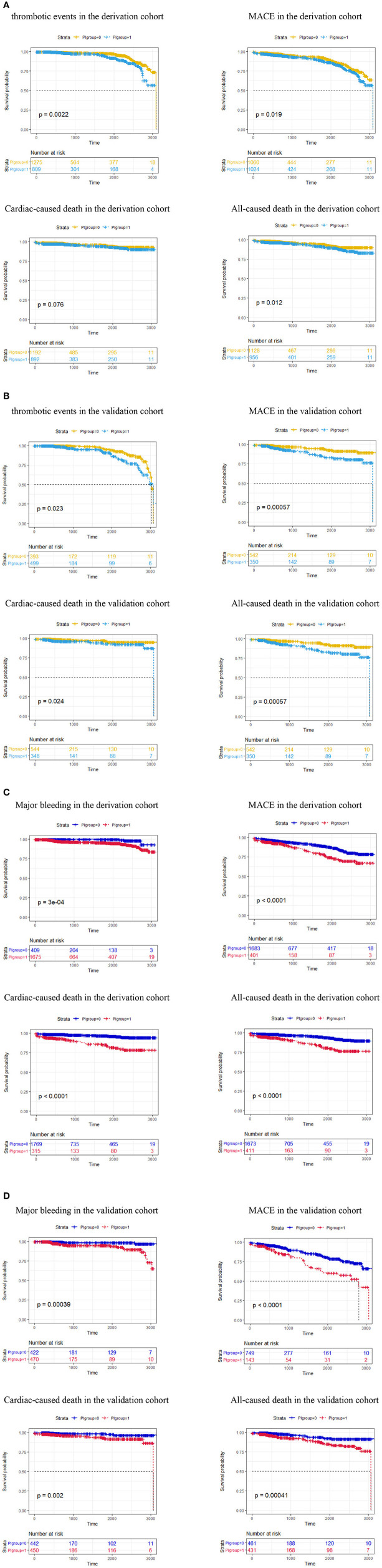
**(A)** Cumulative 3-year incidence of thrombotic events (*p* = 0.0022), MACEs (*p* = 0.019), cardiac-caused death (*p* = 0.076), and all-caused death (*p* = 0.012) according to the thrombotic risk score categories in the derivation cohort (*N* = 2,084). **(B)** Cumulative 3-year incidence of thrombotic events (*p* = 0.023), MACEs (*p* = 0.00057), cardiac-caused death (*p* = 0.0024), and all-caused death (*p* = 0.00057) according to the thrombotic risk score categories in the validation cohort (*N* = 892). **(C)** Cumulative 3-year incidence of major bleeding events (*p* = 0.0003), MACEs (*p* < 0.0001), cardiac-caused death (*p* < 0.0001), and all-caused death (*p* < 0.0001) according to the thrombotic risk score categories in the derivation cohort (*N* = 2,084). **(D)** Cumulative 3-year incidence of major bleeding events (*p* = 0.00039), MACEs (*p* < 0.0001), cardiac-caused death (*p* = 0.002), and all-caused death (*p* = 0.00041) according to the thrombotic risk score categories in the validation cohort (*N* = 892). MACEs, major adverse cardiovascular events; PI group, prognostic index group; PI group = 0 in Figures 3A,B, low risk group classified by the PI of thrombotic risk score categories; PI group = 1 in Figures 3A,B, high-risk group classified by the PI of thrombotic risk score categories; PI group = 0 in Figures 3C,D, low-risk group classified by the PI of major bleeding risk score categories; PI group = 1 in Figures 3C,D, high risk group classified by the PI of major bleeding risk score categories.

The 3-year cumulative incidences of TEs/MB, MACEs, cardiac death, and all-cause death according to the thrombotic risk score categories and MB risk score categories in the derivation and validation cohorts are presented in Figures 2A–D. In the derivation cohort, TEs (*p* = 0.0022), MACEs (*p* = 0.019), and all-cause death (*p* = 0.012) were significantly different between the low-risk and high-risk groups divided by the PI of thrombotic risk score categories (Figure 2A). Analogously, in the validation cohort, TEs (*p* = 0.023), MACEs (*p* = 0.00057), cardiac death (*p* = 0.024), and all-cause death (*p* = 0.00057) were significantly different between the low-risk and high-risk groups divided by the PI of thrombotic risk score categories (Figure 2B). Similar results are presented in Figures 2C,D.

**Figure 3:**
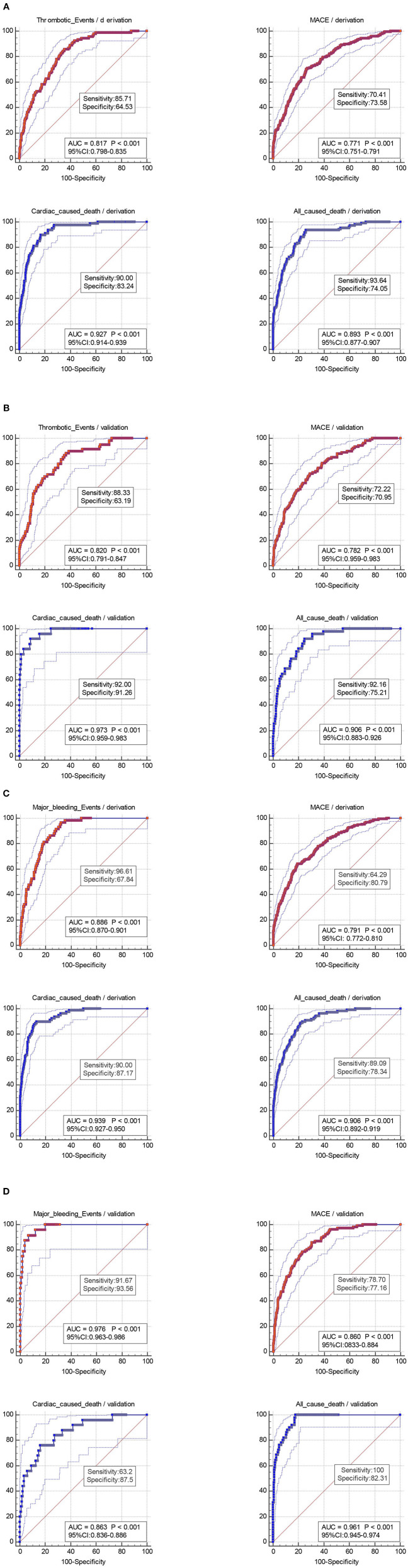
**(A)** ROC curve of thrombotic events (*p* < 0.001), MACEs (*p* < 0.001), cardiac-caused death (*p* < 0.001), and all-caused death (*p* < 0.001) according to the thrombotic risk score categories in the derivation cohort (*N* = 2,084). **(B)** Cumulative 3-year incidence of thrombotic events (*p* < 0.001), MACEs (*p* < 0.001), cardiac-caused death (*p* < 0.001), and all-caused death (*p* < 0.001) according to the thrombotic risk score categories in the validation cohort (*N* = 892). **(C)** Cumulative 3-year incidence of major bleeding events (*p* < 0.001), MACEs (*p* < 0.001), cardiac-caused death (*p* < 0.001), and all-caused death (*p* < 0.001) according to the major bleeding risk score categories in the derivation cohort (*N* = 2,084). **(D)** Cumulative 3-year incidence of major bleeding events (*p* < 0.001), MACEs (*p* < 0.001), cardiac-caused death (*p* < 0.001), and all-caused death (*p* < 0.001) according to the major bleeding risk score categories in the validation cohort (*N* = 892). MACEs, major adverse cardiovascular events.

Figure 3 shows the ROC curves for the discriminatory value of the 3-year evaluation performance of the risk prediction model in the derivation and validation cohorts. The AUCs by the TE prediction project were 0.817, 0.771, 0.927, and 0.893 for TEs, MACEs, cardiac death, and all-cause death, respectively, in the derivation cohort (Figure 3A). The AUCs by the TE prediction project were 0.820, 0.782,0.973, and 0.906 for TEs, MACEs, cardiac death, and all-cause death, respectively, in the validation cohort (Figure 3B). The AUCs by the MB prediction project were 0.886, 0.791, 0.939, and 0.906 for bleeding, MACEs, cardiac death, and all-cause death, respectively, in the derivation cohort (Figure 3C). The AUCs by the MB prediction project were 0.976, 0.86, 0.863, and 0.961 for bleeding, MACEs, cardiac death, and all-cause death, respectively, in the validation cohort (Figure 3D).

### Evaluation of the Risk Prediction Model

**Figure 4:**
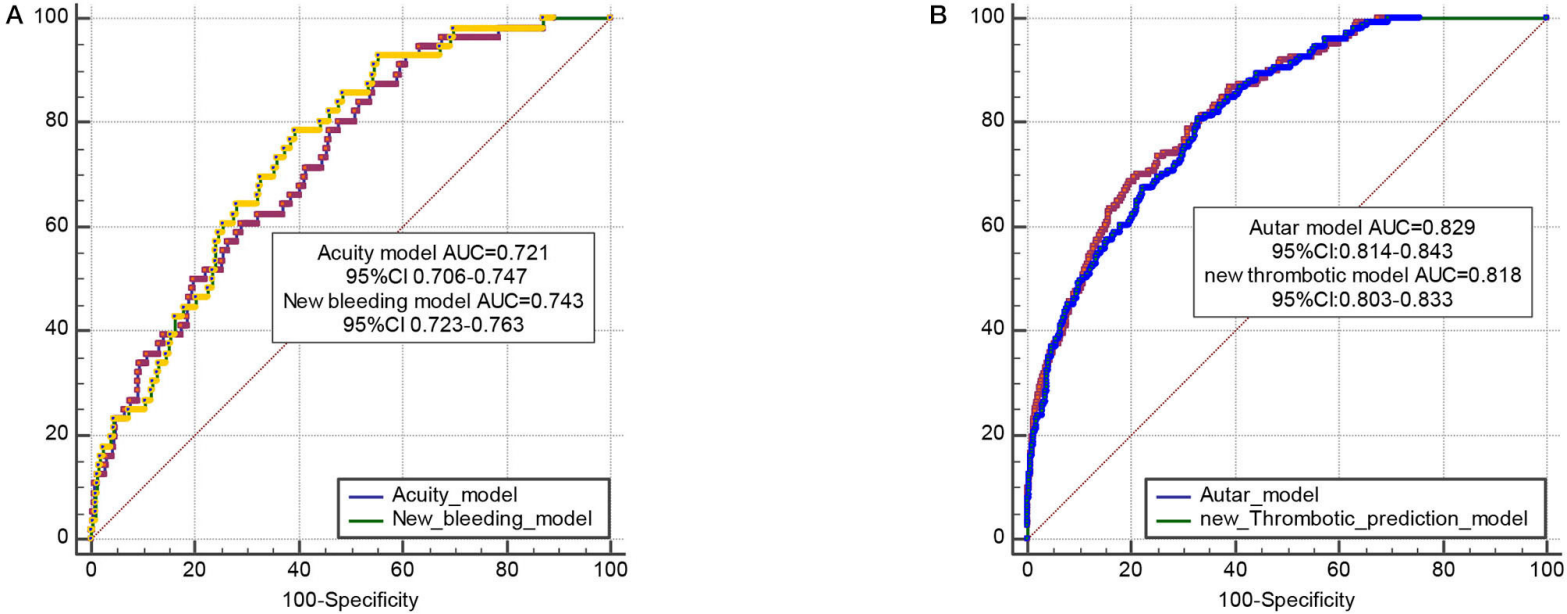
**(A)**. Pairwise comparison of ROC curves between the new bleeding model and the acuity risk score model (*N* = 3,976). The area under the ROC curve (AUC) of the new bleeding model is 0.743 [confidence interval (CI)0.723–0.763], and the AUC of acuity score is 0.721 (CI.706–0.747). *P* = 0.4047, z statistic, 0.833. **(B)**. Pairwise comparison of ROC curves between the new thrombotic model and the autar risk score model (*N* = 3,976). The AUC of new the thrombotic model is 0.818 (CI.803–0.833), and the AUC of acuity score is 0.829 (0.814–0.843). *p* = 0.1079, z statistic, 1.608.

Appendix Figure 1 illustrates the evaluation performed, including the calibration curve (Appendix Figures 1A,B) and decision curve analysis (DCA) curve (Appendix Figure 1C) for TEs and MB, respectively, in the derivation and validation cohorts. It is comparable between the observed and predicted risks projects, illustrating that the model calibration was excellent for both predicting scores. Figure 4A shows a pairwise comparison of ROC curves between the new bleeding model and acuity risk score model. The AUC of the new bleeding model is 0.743, and the AUC of acuity score is 0.721. Figure 4B shows a pairwise comparison of ROC curves between the new thrombotic model and autar risk score model. The AUC of the new thrombotic model is 0.818, and the AUC of acuity score is 0.829.

## Discussion

This study, which involved 2,976 real-world patients with multivessel coronary artery disease who had undergone primary PCI in China, yielded the following main findings: first, we reported the development of separate models for predicting the risks of TEs and MB, which demonstrated moderate accuracy in discrimination concordant and stratified the risk in the derivation and validation cohorts; second, we showed that subjects with high thrombotic risk also had high bleeding risk in a large proportion of the study population.

The objective of this study was to identify readily available characteristics that were independently correlated with TEs and MB in patients with multivessel lesions who had undergone primary PCI. The study described risk indicators (e.g., clinical characteristics, angiography). While various tools have been developed to stratify risk after undergoing PCI, the majority of them are focused on peri-procedural short-term outcomes (6–9, 11, 17–19). We modeled events occurring after discharge and found that clinical risk factors and procedural parameters could predict the risk of TEs. This result is consistent with the findings of previous studies that emphasized the importance of the complexity of coronary artery lesions with respect to the risk of TEs (6, 20). Nevertheless, this result differs from the finding of a previous study that highlighted the importance of clinical risk factors alone (21). These discrepancies may be because thrombotic risk factors are not static but dynamic over time. A thrombotic risk score was proposed in the Thrombin Receptor Antagonist in Secondary Prevention of Atherothrombotic Ischemic Events–Thrombolysis in Myocardial Infarction 50 trial (22), which included ischemic stroke as a TE; age was the common independent predictor of TEs in that previous study. According to the most recent ESC guidelines for the management of patients presenting with NSTEMI, based on the result of the ISAR-REACT 5 trial, prasugrel is the recommended drug in patients who proceed to PCI. No patient in this study assumed prasugrel, because this was not the antithrombotic drug of choice during the period in which the patients were enrolled. Analogously, we identified older age as an independent determinant of long-term TEs, which is consistent with the previously observed association between parameters and thrombosis. It is plausible to include ischemic stroke as one of the components of the TE composite endpoint. Among subjects who underwent PCI, ischemic stroke, which demands intensive antithrombotic therapy, is as clinically important as MI and stent thrombosis.

The risk factors constituting the bleeding risk score established in our study were consistent with those in previous studies generally (2, 7, 23). Not surprisingly, we identified triple therapy [defined as a combination of oral anticoagulant therapy and DAPT (aspirin plus thienopyridine)] at discharge as an independent determinant of long-term bleeding, which is consistent with previous observations (21). Nonetheless, female sex, older age, and renal dysfunction were absent in our final bleeding model. It is possible that earlier studies that focused on shorter-term events accounted for these discrepancies and that underlying risk factors for bleeding were not constant but variable over time resulting in differences. Analogously, the hypothesis is similar to the findings of Genereux et al. (2) and Ducrocq et al. (24), who reported that bifurcation lesions were associated with post-discharge bleeding. However, no prospective study has indicated clinical utility to guide treatment decisions. The use of proton pump inhibitors has been shown to reduce the incidence of gastrointestinal bleeding in patients undergoing PCI, and liberal prophylactic use is essential for the prognosis of these patients. The present prediction project assessing bleeding events was generally consistent with previous studies, and the difference in the risk factors identified might be attributable to the selected population, race, and study design. Therefore, it is preferable to practice the prediction rule derived from the cohort with homologous characteristics of the race. The present prediction project evaluating MB risks showed modest accuracy in both the derivation and validation cohorts. AUCs ranged from 0.791 to 0.976, indicating that the risk score was helpful for discrimination in the clinical prediction models. We performed a pairwise comparison of the new bleeding model with the acuity risk score model, and the new thrombotic model with the autar risk score model. The AUC of the new bleeding model is larger than the acuity score, which showed excellent performance. Although the AUC of the new thrombotic model is similar to the autar risk score, it still simplified the model of predicting thrombosis events and is of benefit for application in clinical assessment.

It is recommended and well-validated by guidelines that risk stratification tools assist with therapeutic decision-making for patients with acute coronary syndrome (25–28). This study included more complex patients than previous trials, that is, this study included patients with two-vessel and triple-vessel diseases. In the context of the growing trend toward individualization and evidence-based therapy, risk stratification could meet patient preferences and enhance patient compliance while balancing against the adverse effects of some therapies (e.g., thrombosis and bleeding) in patients with multivessel disease. The risk stratification strategy outlined in this study provides clinic doctors with an opportunity to select potential candidates with the greatest absolute gains, and it is important to offer therapeutic interventions for secondary prevention of acute MI. We did not include the type of stent used, because previous trials had reported no significant discrepancy in the incidence of TEs between bare-metal stents and first-generation drug-eluting stents (29, 30).

Out-of-hospital stroke results in substantial mortality and morbidity. Considering that substantial mortality and morbidity are correlated with post-PCI ischemic stroke, more studies evaluating risk factors are required to prevent post-PCI ischemic stroke. The Organization to Assess Strategies for Ischemic Syndromes I and II studies (31) reported that stroke in subjects with the coronary disease was correlated with a 6-month mortality rate of 27%. Therefore, we assessed the incidence of stroke in patients who underwent primary PCI. Ischemic stroke and hemorrhagic stroke were categorized as a TE and an MB event, respectively. IABP use and cerebral hemodynamic impairment, especially ischemic stroke, have potential associations. This study assessed risk factors for TEs after primary PCI; however, no association was identified. According to the most recent ESC guideline for the management of patients presenting with NSTEMI, triple antithrombotic therapy is suggested only for 1 week; this recent recommendation has probably reduced the incidence of bleeding.

In this study, we have found that a lower degree of lesion stenosis contributed to MB. After reviewing the literature, we did not find a reasonable explanation for this result. Therefore, basic research on this aspect should be carried out accordingly in the future to carry out relevant research from the perspective of the mechanism. The study of Marco Zimarino et al. (32) has made the conclusion that PCI of bifurcation lesions is associated with increased risk of thrombotic events and investigated the theme of the duration of DAPT after PCI of bifurcation lesion. The literature has exposed the state of art concerning of the choice of antithrombotic drugs, timing of initiation, the DAPT duration, risk stratification and overall the identification of patients at high bleeding risk, with a decision-making algorithm for DAPT duration in PCI in coronary bifurcation.

The PRECISE-DAPT score, which has been validated in two large independent patient populations with ACS, is a five-item bleeding risk prediction model developed to estimate the bleeding risk in patients who receive DAPT after stent implantation (4). Based on the PRECISE-DAPT score, categorization of patients has been proved to be valuable to inform decision-making for the duration of DAPT in stented patients (33, 34). There are two retrospective analyses (35, 36) that showed an absolute bigger reduction of ischemic risk in patients who are receiving long-term DAPT after the complex intervention. Furthermore, patients who have undergone complex intervention simultaneously carry features that greatly increase their bleeding risk, including renal disease, multiple comorbidities, and previous bleeding.

## Study Limitations

This study has several important limitations that should not be ignored. First of all, it has an observational and prospective design, which precludes causal inference and carries inherent limitations. Second, information on the history of previous bleeding events, which could be significantly correlated with exceedingly great risk for MB events, has not been collected. Third, potential reporting bias might have been introduced, accounting for the fact that bleeding events were not adjudicated by a blinded clinical event committee independently. Fourth, the study is derived between 2010 and 2017, and early-generation drug-eluting stents are not distinguished from new-generation drug-eluting stents. Fifth, follow-up information is prospectively recorded according to prespecified definitions, which may limit the power to identify other predictors of stroke. Additionally, genetic characteristics and unidentified biochemical parameters should be considered to provide additional optimization of antithrombotic benefit and reduce bleeding risk. The study population was mainly male, which induced an obvious gender bias. Therefore, a larger prospective study should be needed to explore the separate models for predicting the risks of TEs and MB. This study did not find any correlation between CKD, which is considered as a dichotomous variable in this study, and incidence of bleeding. However, different scores developed in other studies (7, 23) examined not only the presence of renal impairment but also the grade of the dysfunction. Therefore, we might discriminate the grades of chronic kidney disease (stages 3, 4, and 5) in further larger studies. Finally, the cohort in this study was derived from patients who agreed to participate, which might have resulted in unaccounted selection pressures that affect the generalizability of the total cohort.

## Conclusion

This study reported the development of separate models for predicting the risks of TEs and MB in subjects with multivessel coronary artery disease who had undergone primary PCI, which demonstrated moderate accuracy in discrimination concordant and stratified the risk in the derivation and validation cohorts. Furthermore, this study showed that subjects with a high incidence of thrombotic risk had greater bleeding risk in a large proportion of the study population.

## Data Availability Statement

The datasets used and/or analyzed during this study are available from the corresponding author on reasonable request. Requests to access these datasets should be directed to hbyanfuwai2018@163.com.

## Ethics Statement

The studies involving human participants were reviewed and approved by Ethics Committee of Fuwai Hospital. The patients/participants provided their written informed consent to participate in this study.

## Author Contributions

HY, XZ, CL, PZ, ZS, JL, JZ, RC, YW, YC, LS, and HZ: substantial contributions to conception and design, data acquisition, or data analysis and interpretation, drafting the article or critically revising it for important intellectual content, final approval of the version to be published, and agreement to be accountable for all aspects of the study in ensuring that questions related to the accuracy or integrity of the study are appropriately investigated and resolved. All authors contributed to the article and approved the submitted version.

## Funding

This study was supported by the Chinese Academy of Medical Sciences Innovation Fund for Medical Sciences (2016-I2M-1-009), National Natural Science Funds (Number: 81970308), the Fund of Sanming Project of Medicine in Shenzhen (Number: SZSM201911017) and Shenzhen Key Medical Discipline Construction Fund (number: SZXK001).

## Conflict of Interest

The authors declare that the research was conducted in the absence of any commercial or financial relationships that could be construed as a potential conflict of interest.

## Publisher's Note

All claims expressed in this article are solely those of the authors and do not necessarily represent those of their affiliated organizations, or those of the publisher, the editors and the reviewers. Any product that may be evaluated in this article, or claim that may be made by its manufacturer, is not guaranteed or endorsed by the publisher.

## Footnotes

^1^The authors have completed the TRIPOD reporting checklist.
