# Supplementary material for: Thrombosis and Major Bleeding Risk After Primary PCI Among Patients With Multivessel Coronary Artery Disease
Source: Front Cardiovasc Med. 2022 Feb 8;8:729432. doi: 10.3389/fcvm.2021.729432 (PMC8862174; doi:10.3389/fcvm.2021.729432)
Supplement: Supplementary file 1 [file Data_Sheet_1.pdf]

TRIPOD Checklist: Prediction Model Development

Section	Item	Checklist description	Reported on Page Number/Line Number	Reported on Section/Paragraph
**Title and abstract**				
Title	1	Identify the study as developing and/or validating a multivariable prediction model, the target population, and the outcome to be predicted.	Page 1/Line 1	Title/ Para 1
Abstract	2	Provide a summary of objectives, study design, setting, participants, sample size, predictors, outcome, statistical analysis, results, and conclusions.	Page 2/Line 2-21	Abstract/Para 1
**Introduction**				
Background and objectives	3a	Explain the medical context (including whether diagnostic or prognostic) and rationale for developing or validating the multivariable prediction model, including references to existing models.	Page 3/Line 3-15	Background/ Para 1
	3b	Specify the objectives, including whether the study describes the development or validation of the model or both.	Page 3/Line 16-20	Background/ Para 2
**Methods**				
Source of data	4a	Describe the study design or source of data (e.g., randomized trial, cohort, or registry data), separately for the development and validation data sets, ifapplicable.	Page 4/Line 2-8	Material and Methods/Para 1
	4b	Specify the key study dates, including start of accrual; end of accrual; and, if applicable, end of follow-up.	Page 4/Line 5	Material and
Participants	5a	Specify key elements of the study setting (e.g., primary care, secondary care, general population) including number and location of centres.	Page 5/Line 7-9	Material and Methods/Para 2
	5b	Describe eligibility criteria for participants.	Page 4/Line 8-14	Material and
	5c	Give details of treatments received, if relevant.	Page 4/Line 18-22	Material and
Outcome	6a	Clearly define the outcome that is predicted by the prediction model, including how and when assessed.	Page 5/Line 7-9	Definitions/Para 1
	6b	Report any actions to blind assessment of the outcome to be predicted.	Page 6/Line 9-10	Definitions/Para 1
Predictors	7a	Clearly define all predictors used in developing or validating the multivariable prediction model, including how and when they were measured.	Page 5/Line 8-21	Definitions/Para 1
	7b	Report any actions to blind assessment of predictors for the outcome and other predictors.	Page 5/Line 19-20	Definitions/Para 1
Sample size	8	Explain how the study size was arrived at.	Page 4/Line 13-14	Material and

Missing data	9	Describe how missing data were handled (e.g., complete-case analysis, single imputation, multiple imputation) with details of any imputation method.	Page 6/Line 19-20	Statistical analysis/Para 1
Statistical analysis methods	10a	Describe how predictors were handled in the analyses.	Page 7/Line 4-6	Statistical analysis/Para 2
	10b	Specify type of model, all model-building procedures (including any predictor selection), and method for internal validation.	Page 7/Line 14-22	Statistical analysis/Para 3
	10d	Specify all measures used to assess model performance and, if relevant, to compare multiple models.	Page 8/Line 6-20	Statistical analysis/Para 4
Risk groups	11	Provide details on how risk groups were created, if done.	Page 8/Line 6-20	Statistical analysis/Para 4
**Results**				
Participants	13a	Describe the flow of participants through the study, including the number of participants with and without the outcome and, if applicable, a summary of the follow-up time. A diagram may be helpful.	Page 9/Line 1-6	Results/Para 1
	13b	Describe the characteristics of the participants (basic demographics, clinical features, available predictors), including the number of participants with missing data for predictors and outcome.	Page 9/Line 7-13	Results/Para 1
Model development	14a	Specify the number of participants and outcome events in each analysis.	Page 9/Line 9-13	Results/Para 1
	14b	If done, report the unadjusted association between each candidate predictor and outcome.	Page 9/Line 15-20	Results/Para 2
Model specification	15a	Present the full prediction model to allow predictions for individuals (i.e., all regression coefficients, and model intercept or baseline survival at a given time point).	Page 9/Line 15-20	Results/Para 2
	15b	Explain how to the use the prediction model.	Page 10/Line 1-22	Results/Para 3-4
Model performance	16	Report performance measures (with CIs) for the prediction model.	Page 11/Line 1-20	Results/Para 5-6
**Discussion**				
Limitations	18	Discuss any limitations of the study (such as nonrepresentative sample, few events per predictor, missing data).	Page 14/Line 16-22	Limitation/Para 1
Interpretation	19b	Give an overall interpretation of the results, considering objectives, limitations, and results from similar studies, and other relevant evidence.	Page 11-13/Line 1-22	Discussion/Para 1-3
Implications	20	Discuss the potential clinical use of the model and implications for future research.	Page 15/Line 10-16	Conclusion/Para 1
**Other information**				
Supplementary information	21	Provide information about the availability of supplementary resources, such as study protocol, Web calculator, and data sets.	Page 16/Line 4-6	Data Availability/Para 1
Funding	22	Give the source of funding and the role of the funders for the present study.	Page 16/Line 7-9	Funding/Para 1
