# Supplementary material for: Thrombosis and Major Bleeding Risk After Primary PCI Among Patients With Multivessel Coronary Artery Disease
Source: Front Cardiovasc Med. 2022 Feb 8;8:729432. doi: 10.3389/fcvm.2021.729432 (PMC8862174; doi:10.3389/fcvm.2021.729432)
Supplement: Supplementary file 2 [file Data_Sheet_2.docx]

| Appendix Figure1 A |  |
| --- | --- |
| Calibration of 3-year thrombotic events in the derivation cohort | Calibration of 5-year thrombotic events in the derivation cohort |
| 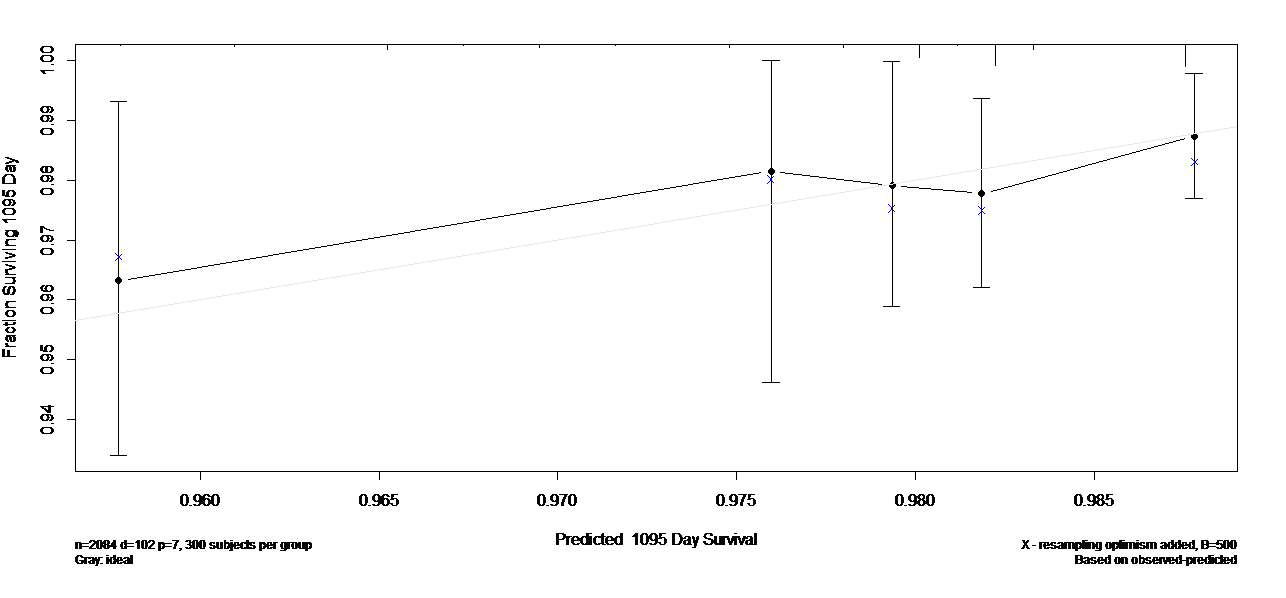 | 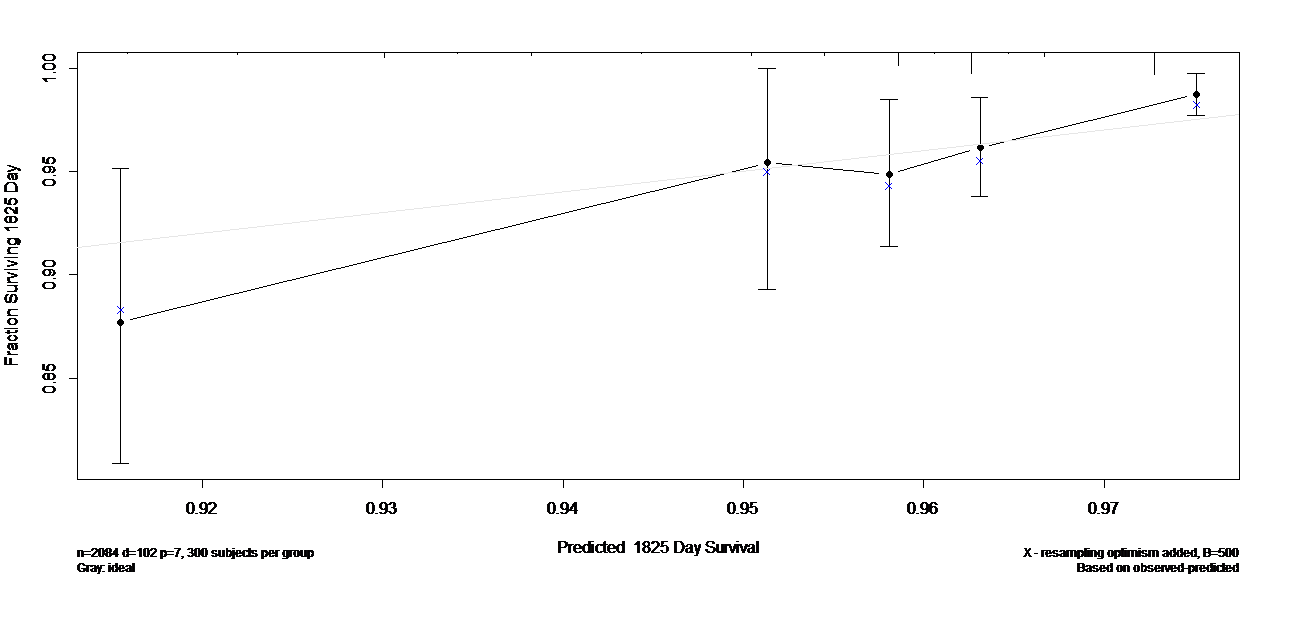 |
| Calibration of 3-year bleeding events in the derivation cohort | Calibration of 5-year bleeding events in the derivation cohort |
| 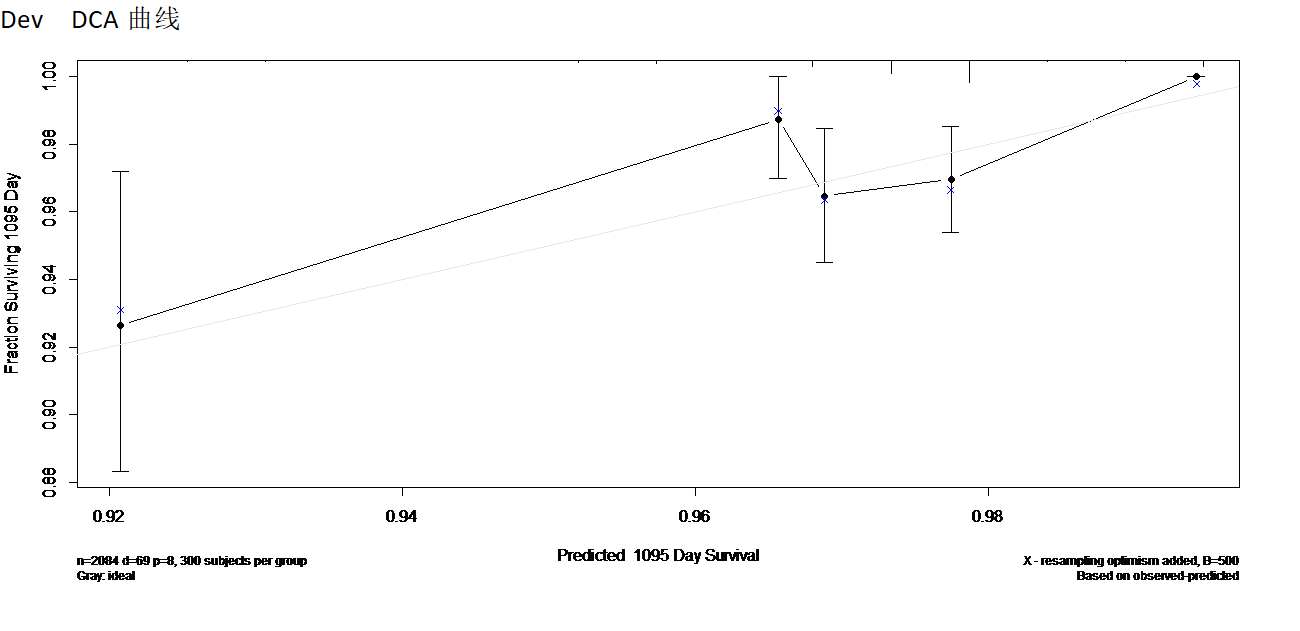 | 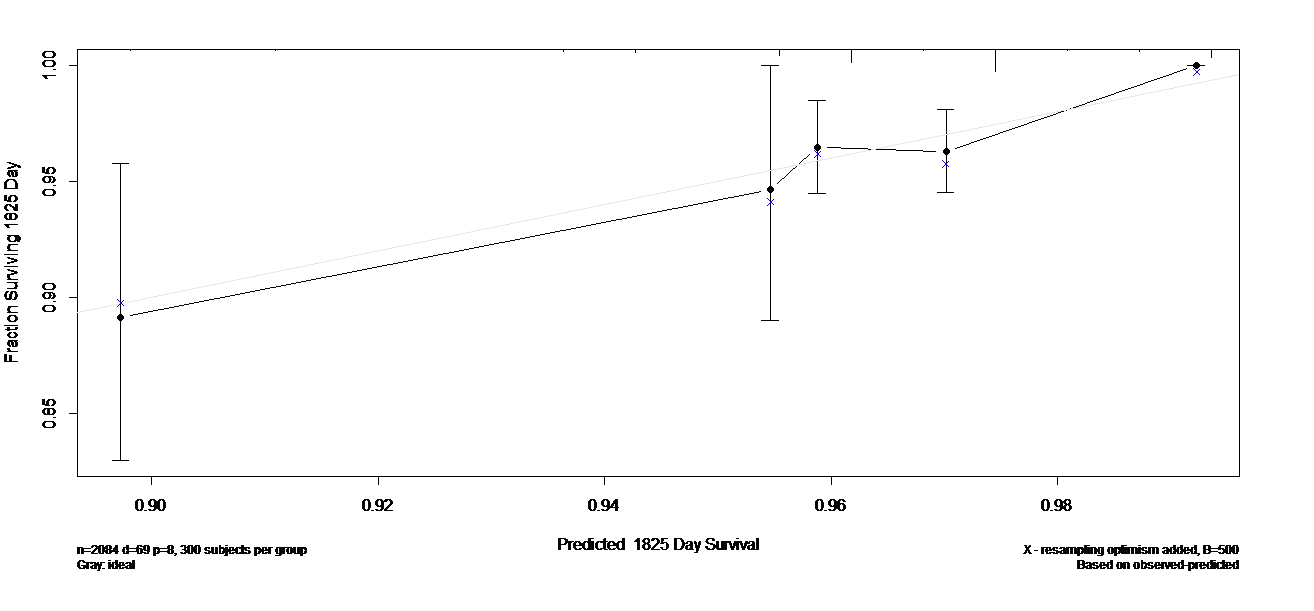 |
| Appendix Figure 1 B |  |
| Calibration of 3-year thrombotic events in the validation cohort | Calibration of 5-year thrombotic events in the validation cohort |
| 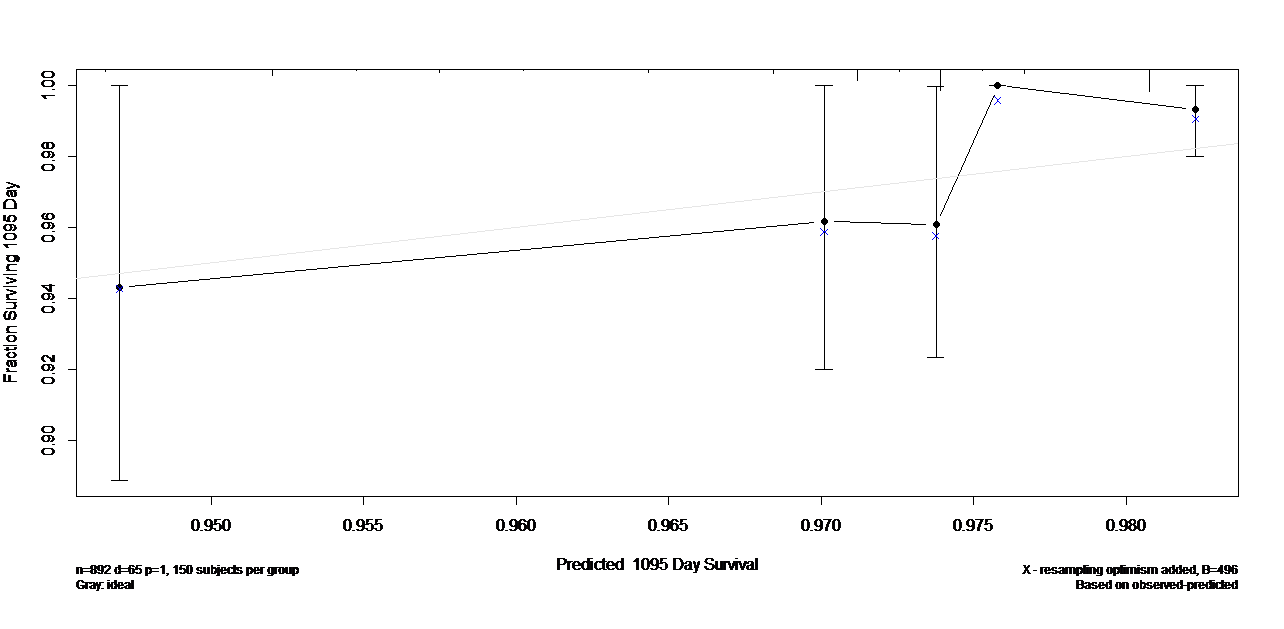 | 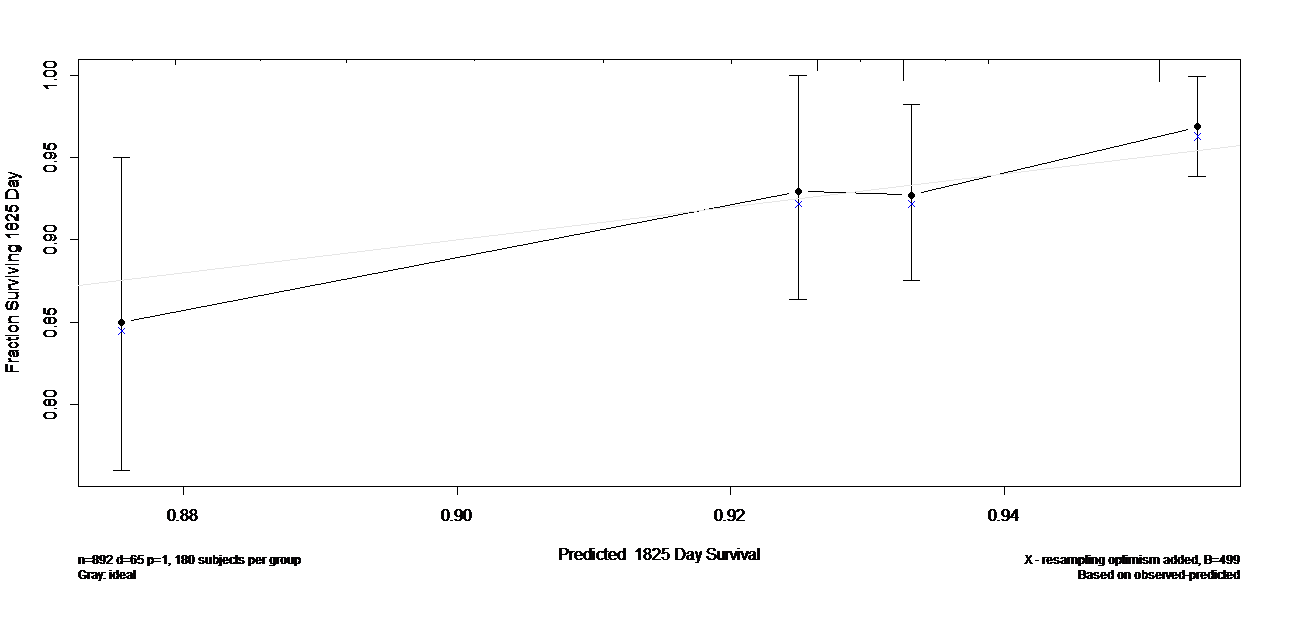 |
| Calibration of 3-year bleeding events in the validation cohort | Calibration of 5-year bleeding events in the validation cohort |
| 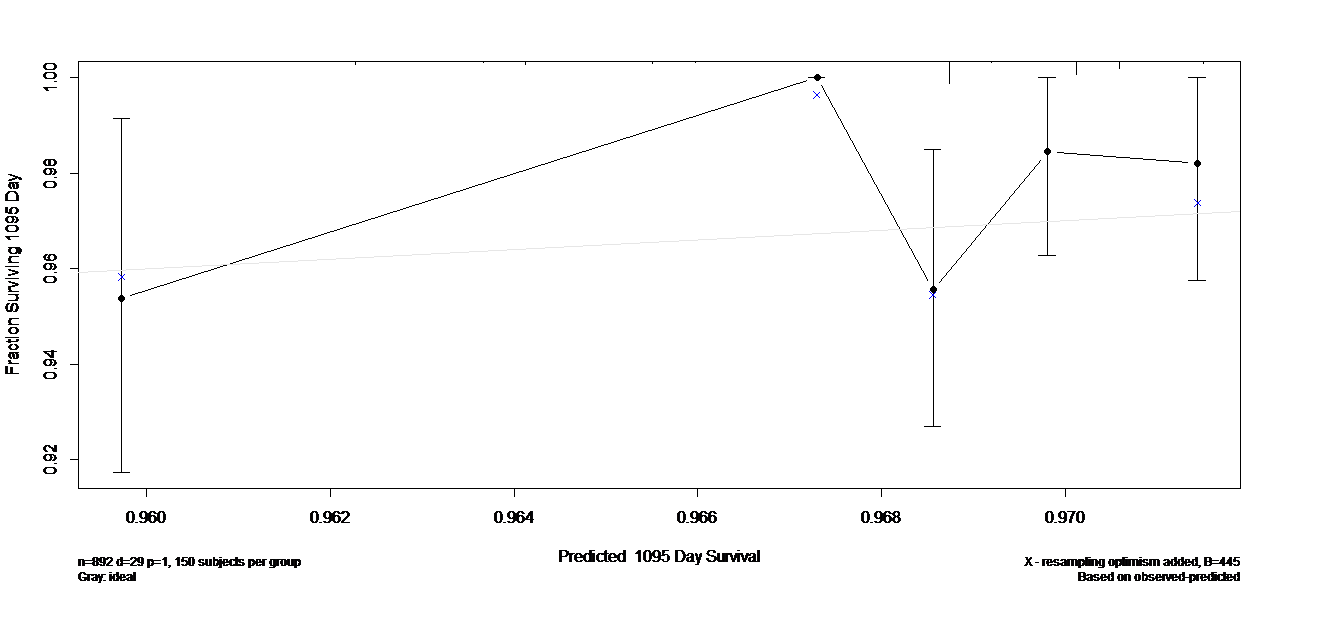 | 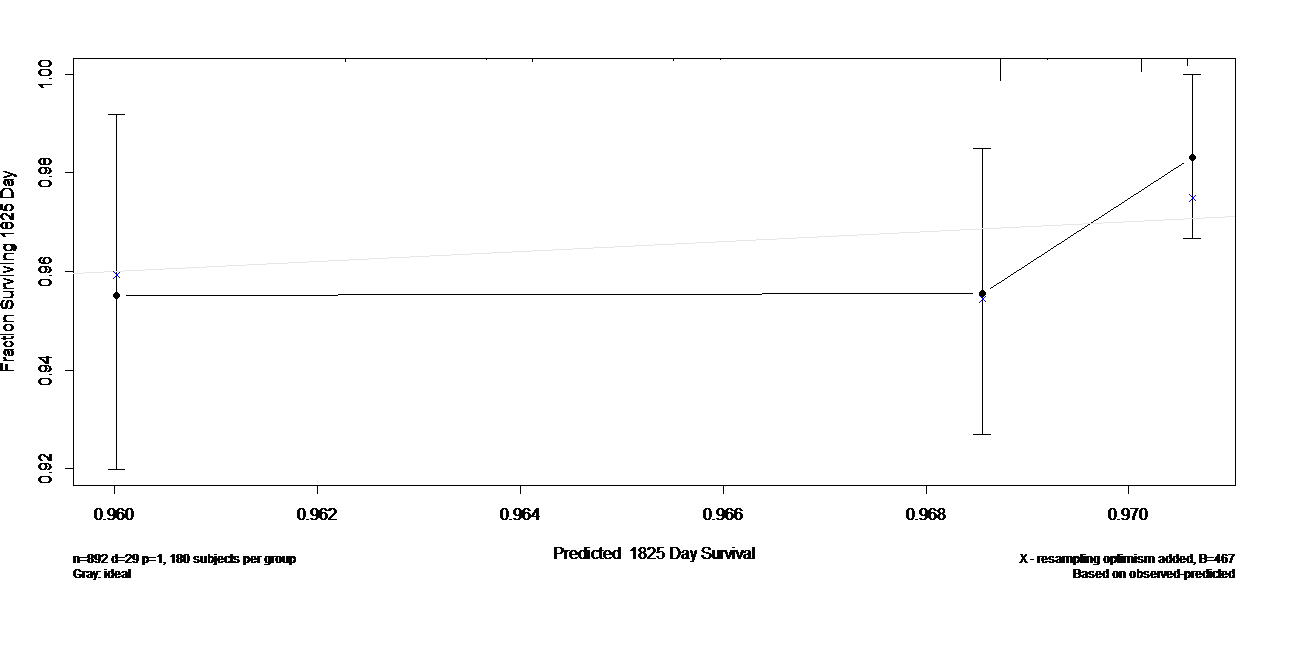 |
| Appendix Figure1 C |  |
| DCA curve of thrombotic events in the derivation cohort | DCA curve of major bleeding events in the derivation cohort |
| 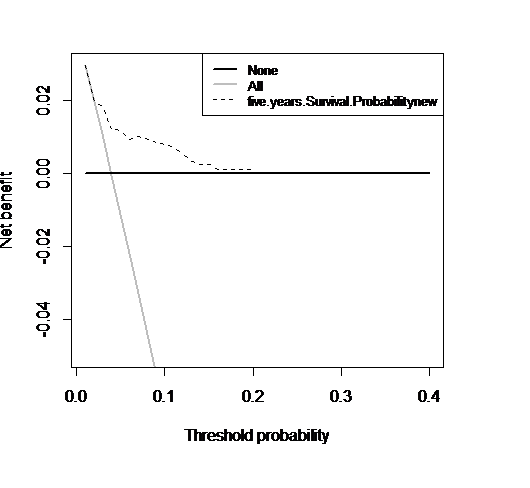 | 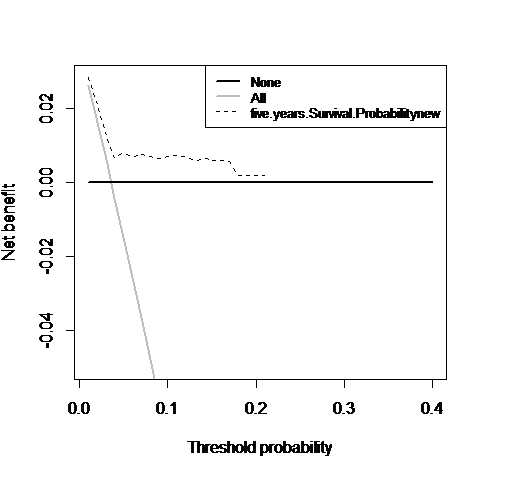 |

Appendix Figure 1

A, Calibration of 3-year and 5-year thrombotic events and major bleeding in the derivation cohort (N=2084).

B, Calibration of 3-year and 5-year thrombotic events and major bleeding in the validation cohort (N=982)

C, DCA curve of thrombotic events and major bleeding in the derivation cohort
